# Myeloperoxidase as a Promising Therapeutic Target after Myocardial Infarction

**DOI:** 10.3390/antiox13070788

**Published:** 2024-06-28

**Authors:** Maxwell Quinn, Richard Y. K. Zhang, Idris Bello, Kerry-Anne Rye, Shane R. Thomas

**Affiliations:** Cardiometabolic Disease Research Group, School of Biomedical Sciences, Faculty of Medicine & Health, University of New South Wales, Sydney, NSW 2052, Australia

**Keywords:** myeloperoxidase, oxidative stress, atherosclerosis, myocardial infarction, left ventricular remodelling, heart failure

## Abstract

Coronary artery disease (CAD) and myocardial infarction (MI) remain leading causes of death and disability worldwide. CAD begins with the formation of atherosclerotic plaques within the intimal layer of the coronary arteries, a process driven by persistent arterial inflammation and oxidation. Myeloperoxidase (MPO), a mammalian haem peroxidase enzyme primarily expressed within neutrophils and monocytes, has been increasingly recognised as a key pro-inflammatory and oxidative enzyme promoting the development of vulnerable coronary atherosclerotic plaques that are prone to rupture, and can precipitate a MI. Mounting evidence also implicates a pathogenic role for MPO in the inflammatory process that follows a MI, which is characterised by the rapid infiltration of activated neutrophils into the damaged myocardium and the release of MPO. Excessive and persistent cardiac inflammation impairs normal cardiac healing post-MI, resulting in adverse cardiac outcomes and poorer long-term cardiac function, and eventually heart failure. This review summarises the evidence for MPO as a significant oxidative enzyme contributing to the inappropriate inflammatory responses driving the progression of CAD and poor cardiac healing after a MI. It also details the proposed mechanisms underlying MPO’s pathogenic actions and explores MPO as a novel therapeutic target for the treatment of unstable CAD and cardiac damage post-MI.

## 1. Introduction

Coronary artery disease (CAD) and myocardial infarction (MI) constitute a significant proportion of the growing burden of cardiovascular disease (CVD) in the world’s ageing population and remains a leading cause of global morbidity and mortality, accounting for >9-million deaths in 2021 [1]. Despite the significant investment of finances, time, and resources in the pursuit of treatments to ameliorate or reverse the progression and clinical manifestations of CAD, the discovery of definitive treatments remains elusive [2]. Accordingly, there is continued interest in identifying the key molecular and cellular processes that drive atherosclerotic lesion rupture in CAD patients and precipitate a MI, and the ensuing aberrant cardiac healing and dysfunction that increases an MI patient’s future risk of developing heart failure (HF).

The current review focuses on the role of the haem peroxidase enzyme myeloperoxidase (MPO), which is highly-expressed in neutrophils, and to a lesser extent in monocytes and discrete tissue sub-sets of macrophages [3]. Active MPO is secreted extracellularly upon leukocyte activation, resulting in the production of a range of reactive oxidants. In the context of infection, MPO-catalysed oxidative reactions contribute to the innate immune defence and the inflammatory phase of wound healing [3]. However, as detailed in this review, considerable evidence has identified a pathogenic role for MPO-derived oxidants in driving the development of vulnerable atherosclerotic lesions in CAD patients and cardiac complications post-MI. Here, we discuss the muti-faceted roles and molecular mechanisms by which MPO influences the progression and clinical outcomes of CAD and MI and the extent to which it represents a viable target for therapeutic intervention. We also discuss the emergence of peroxidasin, a related haem peroxidase family member of MPO, as a potential therapeutic target for treating CAD and MI.

## 2. Inflammation, Oxidative Stress, and Post-MI Ischemia-Reperfusion Injury

CAD is a chronic inflammatory disease of the arterial intima, characterised by the progressive accumulation of cholesterol, immune/inflammatory cells, and fibrotic material that result in the formation of an atherosclerotic plaque [4,5]. Atherosclerosis develops in response to a combination of genetic and modifiable risk factors that include elevated circulating low-density lipoprotein (LDL) cholesterol, diabetes, hypertension, obesity, and smoking [6]. With the persistence of these risk factors, combined with chronic arterial inflammation over decades of life, the advanced fibro-fatty atherosclerotic plaques can become unstable and prone to rupture, forming an occlusive thrombus in the lumen of the coronary artery. The resulting obstruction of oxygenated arterial blood flow to the myocardium precipitates a MI that is characterised by acute cardiomyocyte ischaemia and dysfunction [6]. Prolongation of the ischaemic insult results in cardiomyocyte necrosis, which triggers a complex inflammatory wound healing reaction that eventually replaces the dying cardiomyocytes with non-contractile and collagen-based scar tissue [7]. The degree of damage caused by the ischemic insult and efficacy of the subsequent wound healing process represents important determinants of the risk of MI survivors developing HF in the longer term, with a larger infarct size and a greater area of fibrotic scar tissue predicting an increased risk of HF [8].

In the absence of effective treatment in the acute stages of MI, a wide variety of complications can arise. Within 72 h of coronary artery occlusion, necrotic myocardial tissue can tear and rupture, leading to severe mechanical complications and life-threatening haemodynamic instability, with a mortality rate of approximately 75% [7,9,10]. The most common sites for a rupture include the left ventricular free wall, the interventricular septum, or a papillary muscle, each impairing cardiac function and causing haemodynamic compromise [11,12,13]. Additionally, ischaemic insult to the myocardium can result in the formation of a disrupted and heterogeneous electrical pathway, making the MI survivor prone to potentially life-threatening arrhythmias within hours after the event [10]. Furthermore, poor left ventricle (LV) contraction or LV dysfunction post-MI leads to adjacent intracavitary blood stasis, increasing the risk of large mural thrombus formation, which may subsequently embolise and cause an infarct of distant organs (e.g., the brain, resulting in a stroke) [14]. Thus, rapid clinical management of the MI patient is not only critical for preventing long-term cardiac complications, but also to mitigate acute life-threatening events.

Reperfusion of the ischemic myocardium post-MI to rapidly re-establish arterial blood flow and oxygen supply is the gold standard treatment. However, this can paradoxically increase total myocardial tissue death by up to 50% via a process known as myocardial ischaemia-reperfusion injury (I/RI) [9,10,15]. Lethal reperfusion injury (LRI), the predominant mechanism of myocardial I/RI, is defined as the damage or death to ischaemic, but still viable, cardiomyocytes as a result of reperfusion [16]. LRI was initially considered as simply an ongoing extension of the cellular damage that resulted from the period of ischaemia [17]. However, recent studies have shown that the overall size of an infarct can be reduced with an appropriate LRI-targeted intervention, such as the pharmacological or genetic inhibition of cyclophilin D, at the start of the reperfusion period [18,19,20,21,22]. Cyclophilin D is a core component of the mitochondrial permeability transition pore (MPTP), whose significance to LRI will be discussed further below. While certain LRI-targeted interventions have proven successful in animal models, they have not been effectively translated into the clinical setting [19,20]. Hence, LRI is considered a significant mediator of cardiomyocyte injury and death independent of the initial ischaemic insult, which requires a separate targeted and effective therapeutic intervention. 

While several mechanisms have been proposed to mediate LRI, inflammation and oxidative stress play significant roles [23]. The best understood oxidative mechanism involves the reactivation of the mitochondrial electron transport chain during reperfusion and re-oxygenation, which elevates the generation of reactive oxygen species (ROS) by the organelle [24]. The excessive generation of ROS not only induces aberrant redox cell signalling and local oxidative biomolecule damage, but also activates cyclophilin D. This opens the MPTP, a non-selective channel that resides in the inner mitochondrial membrane [24]. MPTP opening, which only occurs during the reperfusion period, results in ATP depletion and cardiomyocyte death due to mitochondrial membrane depolarisation and the uncoupling of oxidative phosphorylation [25,26]. 

Oxidative stress and resultant myocardial tissue damage during MI also involves other sources of ROS and other molecular mechanisms including the rapid infiltration of activated neutrophils into the infarct zone during the post-MI reperfusion period [27]. These infiltrating neutrophils can degranulate and locally release MPO, a haem peroxidase that catalyses potentially deleterious oxidative reactions including the production of several potent tissue damaging oxidants (e.g., hypochlorous acid [HOCl], hypothiocyanous acid [HOSCN]) and consumption of the important cardioprotective molecule, nitric oxide (NO) [28,29]. These MPO-catalysed oxidative reactions therefore remove the critical homeostatic cardiovascular signalling molecule NO and oxidatively modify cardiomyocyte proteins and lipids that can induce cellular dysfunction and death [3,30]. Below, we briefly introduce the biochemical properties and physiological roles of MPO and provide an overview of the enzyme’s role in the development of atherosclerotic CAD, followed by a focus on MPO’s role in I/RI during MI and heart failure. Finally, we discuss the extent to which targeting MPO represents a potential new therapeutic treatment for the clinical management of MI.

## 3. Biochemistry of MPO

MPO is a member of a broader family of mammalian haem peroxidase enzymes that include eosinophil peroxidase (EPO), peroxidasin (also referred to as vascular peroxidase [VPO-1]), lactoperoxidase (LPO), and thyroid peroxidase (TPO) [31]. EPO and LPO are known for their bactericidal effects, being produced by eosinophils and secretory glands, respectively [32]. TPO is required to produce key thyroid hormones [32,33]. As discussed later, peroxidasin plays a role in vascular wall development and possibly cardiovascular inflammation and disease [34].

MPO is highly expressed in the azurophilic granules of neutrophils [35,36]. Physiologically, MPO plays a crucial role in the innate immune response against invading microbial pathogens through its capacity to generate microbicidal oxidants, including HOCl and HOSCN [36]. At tissue sites of infection or inflammation, MPO is released in large amounts from degranulating activated neutrophils [3]. MPO is also released by activated monocytes, albeit in smaller quantities [37]. While MPO expression is generally downregulated during monocyte differentiation into macrophages, its expression can be preserved in some sub-sets of tissue macrophages upon exposure to cytokines (e.g., granulocyte macrophage colony-stimulating factor, GM-CSF) and pro-atherogenic stimuli [38]. 

Upon release by activated leukocytes, active MPO has the potential to catalyse several oxidative reactions [31] that, as discussed later, are prominent during the sterile inflammatory response to the post-MI LRI phase and contribute to cardiac dysfunction [23].

**Figure 1:**
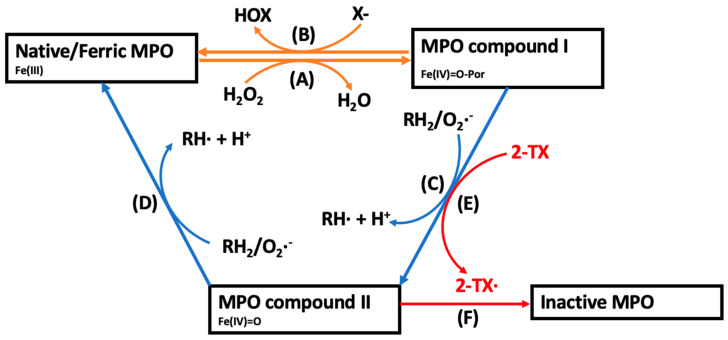
**The catalytic cycles of MPO and the mechanism of action of 2-thioxanthines for irreversible inhibition of MPO.** (**A**) In the presence of H_2_O_2_, native ferric-haem MPO is oxidised to MPO compound I, that, depending on substrate availability, enters the halogenation or peroxidase cycles. (**B**) In the halogenation cycle, compound I mediates the two-electron oxidation of a halide ion (X^−^) into a hypohalous acid (HOX), reforming native MPO. (**C**,**D**) In the peroxidase cycle, MPO compound I undergoes two consecutive one-electron oxidation reactions with various physiological small molecule substrates (RH_2_) forming consecutive diffusible substrate radicals (RH^•^) and reforming the native enzyme [31]. The peroxidase cycle is characterised by the formation of MPO compound II, a reactive intermediate containing a ferryl-oxo (Fe^IV^=O) haem. (**E**) 2-Thioxanthines are rapidly oxidised by compound I into a reactive drug-derived radical (2-TX^•^) [41]. (**F**) The thioxanthine-derived reactive radical irreversibly modifies MPO compound II into an inactive form [41].

In its resting state, MPO’s active site haem exists in its ferric-iron (Fe^III^) form. Upon reaction with its co-substrate, the ROS hydrogen peroxide (H_2_O_2_), the ferric-haem undergoes a two-electron oxidation to form MPO compound I, a powerful oxidant that consists of a ferryl-oxo (Fe^IV^=O) haem and a highly reactive porphyrin π-cation free radical (Por^•+^) (Figure 1, reaction A) [31]. The increased production of the co-substrate, H_2_O_2_, by endothelial cells, smooth muscle cells, and activated leukocytes during vascular disease is vital for maintenance of the enzymatic activity of MPO [39,40]. From this pivotal point, depending on substrate bioavailability, MPO compound I enters either the halogenation or peroxidase cycles [31].

In the halogenation cycle, MPO compound I mediates the two-electron oxidation of several halides (Cl^−^, Br^−^, I^−^) or the pseudohalide, thiocyanate (SCN^−^), into the corresponding hypohalous acid (Figure 1, Reaction B) [31]. At physiological concentrations, Cl^−^ (present at approximately 100 mM) and SCN^−^ (50–200 μM) each account for >50% of H_2_O_2_ consumption catalysed by MPO, while the other halide ions contribute to a lesser extent [42,43]. As such, the implications of HOCl and HOSCN have received considerable attention in the context of cardiovascular disease, as discussed below [31]. 

In the peroxidase cycle, MPO compound I mediates one-electron oxidation reactions with small molecule substrates, including the conversion of the amino acid tyrosine into reactive tyrosyl radicals, NO into nitrite (NO_2_^−^), or NO_2_^−^ into the nitrogen dioxide radical (^•^NO_2_) (Figure 1, reaction C). The resulting MPO compound II (Fe^IV^=O) can mediate a second one-electron oxidation of these substrates and the formation of further radical products, returning the enzyme to its native state (Figure 1, reaction D). Importantly, some of the MPO-derived diffusible substrate radicals that are formed during the peroxidase cycle such as ^•^NO_2_ and tyrosyl radicals can elicit local oxidative tissue damage by oxidizing LDL lipids or inducing endothelial cell and cardiomyocyte damage [44,45,46,47]. Additionally, the catalytic consumption or removal of NO compromises endothelial function in the coronary circulation and increases cardiovascular risk [48,49,50]. The oxidative mechanisms by which MPO can exacerbate cardiovascular disease are discussed in more detail below. 

## 4. MPO in Coronary Artery Disease

### 4.1. Clinical Evidence Correlating MPO with Worsened CAD Outcomes

Clinical evidence has established that circulating levels of MPO are elevated in CAD patients. For example, two case–control studies found that circulating MPO was significantly higher in CAD patients compared to their healthy counterparts [51,52]. Additionally, an increased level of circulating MPO predicts the development of more advanced coronary atherosclerosis, an increased propensity of stable CAD progressing to unstable acute coronary syndrome (ACS) and an increased risk of long-term cardiovascular mortality in patients with established CAD [52,53,54,55,56,57,58,59,60]. Elevated circulating MPO levels may also predict the future development of CAD in otherwise healthy individuals [61] and are associated with worsened prognosis and increased future MI risk in CAD patients [53,62]. Rebeiz et al. found that in patients presenting with chest pain, but without ST-segment elevation nor elevated troponin-T levels, increased MPO levels were strongly associated with an elevated incidence of coronary stenosis (≥70% luminal narrowing), plaque ulceration, thrombus burden, and subsequent percutaneous coronary intervention (PCI) [63]. From this, the measurement of MPO levels has been proposed as an informative clinical biomarker to help identify patients at high risk of experiencing a future ACS event [63]. Regarding the cellular source of elevated circulating MPO in CAD patients, Buffon and colleagues reported that elevated trans-coronary neutrophil activation and the release of MPO occurs more often in CAD patients with unstable versus stable disease [64]. We refer the reader to a recent review that provides a more comprehensive summary of relevant clinical studies examining an association between MPO with cardiovascular outcomes [65].

However, it is important to note that results on the association of circulating MPO levels and CAD risk are conflicting. For example, in patients undergoing elective cardiac angiography, one study found no elevation of plasma MPO in those with stable asymptomatic CAD [66]. The Ludwigshafen Risk and Cardiovascular Health (LURIC) study also reported no difference in circulating MPO levels between patients with or without CAD [67]. However, in that study, circulating MPO levels were independently associated with risk for total and cardiovascular mortality in patients with established CAD [67]. Therefore, while most of the literature indicates an association between circulating MPO levels and degree of CAD progression and clinical cardiovascular risk, the conflicting results from these population-based studies questions the utility of MPO as a routine diagnostic measurement to assess cardiovascular risk. Importantly, accurate and routine measurement of MPO requires careful and rapid isolation and specialised handling of patient blood to avoid ex vivo neutrophil degranulation, as well as the introduction of a routine standardised MPO assay that can be employed across labs. Collectively, these limitations pose significant barriers to the routine clinical use of MPO as a clinical biomarker of CAD progression and cardiovascular risk.

In addition to elevated circulating MPO levels in CAD patients, increased levels of active MPO and biomarkers indicative of oxidative tissue damage by MPO-derived oxidants are found in human atherosclerotic lesions of CAD patients, thereby identifying MPO as a source of oxidative stress in diseased arteries [68,69]. Within established atheroma, MPO is detected in macrophages and extracellularly at the margins of the necrotic core and throughout the sub-endothelium [38,53,70,71,72]. MPO in the subendothelial space is thought to be present due to the transcytosis of circulating MPO across the arterial endothelium [73,74,75]. Biomarkers indicative of MPO-catalysed oxidative reactions in human atherosclerotic plaques include increased levels of HOCl-oxidised proteins, 3-chlorotyrosine, 3-nitrotyrosine, oxidolyl alanine, protein carbamylation, and chlorinated lipids such as α-chloro fatty acid aldehydes and unsaturated lysophopsholipids [69,76,77,78,79]. The levels of MPO and HOCl-oxidised proteins in human lesions correlate with lesion severity and size [71,80]. Moreover, within human atherosclerotic lesions, apoB100 in LDL and apolipoprotein A-I (apoA-I) in HDL are prominent protein targets for MPO-derived oxidants [77,78,81,82,83]. Circulating levels of these apolipoproteins with characteristic MPO-modified amino acids such as carbamyllysine, 3-chlorotyrosine, 3-nitrotyrosine, and oxidized tryptophan are elevated in CAD patients, and correlate with disease severity and risk [77,78,79]. Furthermore, emerging evidence suggests a role for MPO in the formation of neutrophil extracellular traps (NETs) that are linked to plaque instability in both human and animal atherosclerotic plaques [84,85]. 

### 4.2. Preclinical Evidence for MPO as a Driver of Atherosclerosis

Due to the presence of active MPO enzymes in human atherosclerotic lesions, MPO-catalysed oxidative reactions are considered to play a significant role in atherogenesis [86]. Animal studies have provided insights into the role of MPO in experimental atherosclerosis. Early work has reported that MPO gene deficiency either increased or did not change atherosclerotic lesion development in high-fat diet fed LDL-receptor or apolipoprotein E (ApoE) gene-knockout mice, respectively [87]. However, these atherosclerotic lesions in mice did not contain detectable MPO or associated oxidation biomarkers. Similarly, MPO was not found in mouse lesion macrophages, despite being a prominent MPO-expressing cell type in human lesions [38,86]. As mouse leukocytes express ~70% less MPO than human cells, subsequent studies employed transgenic mice that overexpressed the human MPO gene in monocyte/macrophages. This increased detectable MPO in murine lesions and augmented atherosclerosis development by two-fold [88,89]. More recent work utilising the apoE gene-knockout mouse tandem stenosis model of atherosclerotic lesion rupture reported that MPO enzyme activity was elevated in unstable mouse lesions and that loss of the MPO gene or administration of a clinically-trialled MPO inhibitor drug (2-thioxanthine) elevated lesion stability [90]. Together, these pre-clinical animal studies support a proatherogenic role of MPO in lesion macrophages and unstable plaques.

### 4.3. Pro-Atherogenic Mechanisms of MPO

A large body of work has investigated the potential mechanisms by which MPO can promote disease. Several key mechanisms have been identified, including endothelial dysfunction, LDL and HDL oxidation, and matrix metalloproteinase (MMP) activation. Mechanisms of MPO independent of its enzymatic activity are also emerging in the literature.

**Figure 2:**
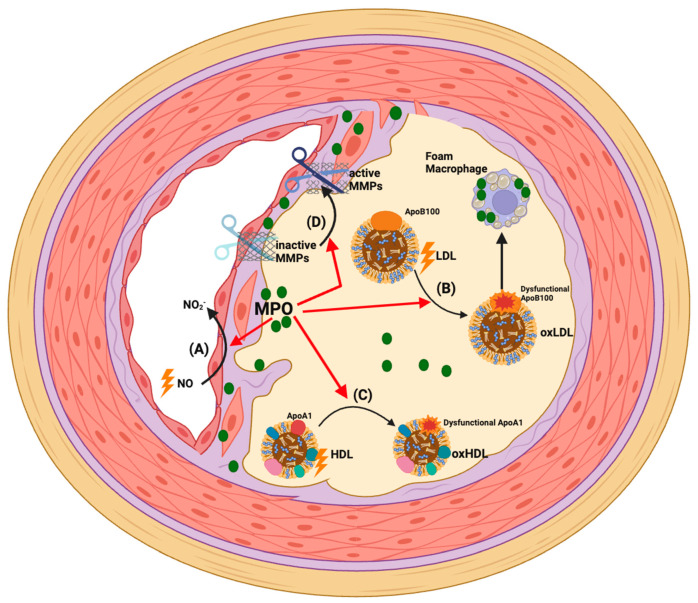
**Pathophysiological actions of MPO in CAD.** Clinical and experimental data support that MPO mediates oxidative stress in atherosclerotic plaques. This accelerates disease development and severity via the following mechanisms: (**A**) Accumulation of MPO in the endothelium and MPO-mediated impairment of NO bioactivity causes endothelial dysfunction and compromises the coronary artery circulation [48,62,70,101]; (**B**) MPO-derived oxidants can directly oxidise apoB100 and LDL lipids such that the modified lipoprotein is recognised by macrophage scavenger receptors. This results in lipid accumulation in macrophages and formation of pro-inflammatory and pro-atherogenic foam cells [102,103,104,105]; (**C**) MPO-derived oxidants can directly oxidise apoA-I in HDL. This impairs HDL’s various cardioprotective functions and generates a pro-inflammatory/dysfunctional lipoprotein particle [78,106]; (**D**) MPO-derived HOCl can promote plaque instability by activating MMPs that proteolytically degrade matrix components in the fibrous cap and may cause plaque rupture [107].

Endothelial Dysfunction: As indicated above, circulating extracellular MPO derived from activated leukocytes can be sequestered into the coronary artery endothelium [64,91,92]. Thus, MPO and its oxidants have been detected in the endothelial and sub-endothelial compartments of diseased human coronary arteries [48]. The accumulation of MPO in the endothelium is thought to primarily involve the attraction of positively charged MPO to negatively charged structures such as heparan sulphate proteoglycans that are expressed on the luminal surface of the arterial endothelium [73,93,94]. Surface-bound MPO is subsequently transcytosed through endothelial cells, and deposited in the sub-endothelial space, where it catalyses oxidative reactions that impair NO bioactivity and promote endothelial dysfunction and vasoconstriction (Figure 2A) [49,50,92,95]. Consistent with this, CAD patients accumulate circulating MPO in the vascular endothelium of the artery, with the degree of vascular sequestration correlating with impaired endothelial function and increased atherosclerotic lesion burden [96]. Moreover, elevated circulating MPO levels in CAD patients independently predict endothelial dysfunction, measured as impaired flow- [97] or acetylcholine-mediated [48] dilation of the brachial artery. Moreover, nicotine infusion (which activates MPO release from circulating neutrophils) can acutely impair endothelial function in healthy control subjects but not in individuals with reduced MPO expression/activity [74]. Pre-clinical studies provide additional evidence of a role for MPO as a mediator of endothelial dysfunction. Eiserich et al. found that MPO knockout mice have preserved vasorelaxation of the aortic endothelium compared to wild-type mice when exposed to endotoxin [75]. Similarly, intravascular administration of MPO into anaesthetised pigs significantly decreased myocardial blood flow and impaired arterial vasorelaxation responses [74]. MPO gene-deficiency also protected against endothelial dysfunction when apoE knockout mice were fed a high-fat diet for 8 weeks [98]. Ex vivo addition of MPO alone or MPO-bound red blood cells has additionally been reported to compromise the NO-dependent relaxation of isolated aortic rings, while in vivo intravascular infusion of MPO increased systemic vascular resistance [99]. MPO has also been identified as a significant cause of pulmonary arteriole constriction and subsequent hypertension under hypoxic conditions in a murine model of pulmonary artery hypertension [100].

Lipoprotein Oxidation: MPO is implicated as a principal source of oxidants that modify and convert LDL into a high-uptake form that is recognised by macrophage scavenger receptors, leading to macrophage foam cells that play a central role in driving the progression of atherosclerosis (Figure 2B) [102,103,104,105]. A further major binding partner and oxidative target of MPO are HDLs, the levels of which are inversely correlated with the progression of CAD and the incidence of ischaemic cardiovascular diseases, including MI [108,109,110,111]. HDLs have several cardioprotective functions including their capacity to mediate reverse cholesterol transport (whereby excess cholesterol is removed from macrophages in the artery wall and peripheral tissues and transported to the liver for excretion in bile [112]). HDLs also have anti-inflammatory, anti-oxidative, anti-apoptotic and endothelial protective functions [113,114,115]. 

ApoA-I, the most abundant HDL apolipoprotein, mediates many of these cardioprotective functions [116]. MPO avidly binds to and oxidises select amino acid residues in apoA-I, which impairs the cardioprotective effects of HDLs (Figure 2C) [117]. Elevated levels of HOCl-oxidised apoA-I have been reported in human atheroma, verifying that MPO targets this apoprotein [77,82,118,119,120,121]. Mass spectrometry has shown that the MPO-mediated site-specific oxidation of tyrosine, methionine, lysine, and tryptophan residues in apoA-I is linked to the functional impairment of HDLs [77,78,106,122,123]. Moreover, MPO-mediated oxidative damage also concomitantly converts HDLs into pro-inflammatory particles that elevate the expression of leukocyte adhesion molecules on endothelial cells [78,124,125,126,127].

MMP Activation: In addition to plaque development, there is growing clinical and experimental evidence indicating that MPO may precipitate atherosclerotic plaque rupture or erosion leading to an ACS [128]. MPO and its oxidation products are enriched in macrophages present in the shoulder regions of unstable human atherosclerotic plaques [38,129]. A high level of MPO-expressing cells is also present in eroded human plaques, suggesting a potential role for MPO in promoting the erosion of the coronary artery endothelium [130]. Furthermore, in human lesions, MPO co-localises with MMPs, which are activated by MPO-derived oxidants, thereby contributing to the degradation of extracellular matrix in the fibrous cap and plaque instability (Figure 2D) [107]. As indicated above, studies in the mouse tandem stenosis model of atherosclerotic plaque instability provides experimental evidence for a causal role of MPO in plaque rupture [131].

Non-Enzymatic Actions of MPO: While less studied, it is important to acknowledge the possible roles of the reported non-enzymatic actions of MPO, which could conceivably also promote cardiovascular inflammation. For example, due to its highly cationic nature, circulating MPO has been reported to preferentially interact with anionic structures such as the heparan sulphate-rich endothelial glycocalyx, which can lead to a collapse of the glycocalyx and shedding of key protein components (e.g., syndecan-1), [132] and reduction in the negative leukocyte-repulsive charge imposed by an intact glycocalyx [94]. The net effect of these physical non-catalytic actions of MPO is to enhance endothelial–leukocyte interactions. Interestingly, recent work by Teo et al. found that increased MPO levels correlated with increased circulating levels of soluble glycocalyx components in COVID-19 patients, and that in vitro inhibition of MPO leads to reduced endothelial shedding of syndecan-1 [133]. MPO may also elevate endothelial–leukocyte interactions by stimulating the endothelial expression of IL-6 and IL-8 [134] or via activating calpain-dependent signalling pathways and inhibition of endothelial NO production [135]. 

MPO may also engage its non-enzymatic action to elevate cardiovascular inflammation through the activation of neutrophils and macrophages. Thus, catalytically-inert MPO can bind cell-surface expressed Mac-1 integrins (CD11b/CD18) to activate neutrophil ROS production and degranulation [136] and retard neutrophil apoptosis to prolong inflammatory responses [137]. Inactive MPO has also been reported to augment macrophage of pro-inflammatory cytokine (e.g., TNFα) and ROS production and improve the phagocytosis and clearance of microbes [138,139,140]. 

The extent to which these various non-catalytic actions are involved in the progression of CAD and post-MI cardiac complications are, however, unknown and warrant further investigations. Importantly, should these non-enzymatic actions of MPO play deleterious roles in CVD, these actions would be refractory to the treatments that target the enzymatic activity of MPO and hence require alternative therapeutic strategies to the MPO inhibitors discussed in the section below.

## 5. Actions of MPO Post-MI

### 5.1. Role of Neutrophils in MI 

MPO is implicated not only in the formation of vulnerable atherosclerotic lesions and their rupture, but it is increasingly considered to be a key contributor to the post-MI inflammation that causes cardiac fibrosis and impairs cardiac function [10]. 

Neutrophils are the major MPO-expressing cells and the first leukocytes to arrive at the infarct site on days 1–3 post-MI. They play a vital role in the initial acute inflammatory response [128,141] by phagocytosing cellular debris from dying cardiomyocytes [27]. However, the pro-inflammatory, proteolytic and pro-oxidant properties of neutrophils during this early phase post-MI can also be detrimental [142] and drive further myocardial injury by secreting proinflammatory cytokines, chemokines, MMPs, and ROS-generating enzymes, especially MPO [7,128,143].

Clinical evidence indicates that neutrophil activation occurs early after MI onset in patients, resulting in the release of ROS and MPO [142]. In plasma samples taken from MI patients, an elevated neutrophil count or neutrophil to lymphocyte ratio upon initial admission is associated with an increase in major adverse cardiac events, as well as an overall poorer short- and long-term prognosis [144,145]. This adverse relationship is independent of key cardiovascular risk factors, including age, tobacco use, diabetes mellitus, hypertension, and hyperlipidaemia [144]. 

Preclinical studies have further elucidated the damaging role of neutrophils post-MI. In animal models of MI, neutrophil extravasation into cardiac tissue disrupts tight cellular junctions and promotes post-ischaemic microvascular leakage [146]. Inhibition of neutrophil infiltration via endothelial-specific deletion of brahma-related gene-1 (Brg1) has significantly reduced infarct volume, left ventricular fibrosis, and the recovery of cardiac function post-I/R injury [147]. Other studies have shown that the blockade of neutrophil infiltration in mice ameliorates cardiac damage and infarct size following reperfusion after surgically-induced MI [148,149,150,151,152,153]. Additionally, neutrophil infiltration and NET formation are amplified in apoE^−/−^ mice post-MI [154]. NETs are characterised by extracellular DNA strands comprised of histones and neutrophilic granule proteins (including MPO), and are part of the innate immune system, ensnaring pathogens and promoting inflammation [155,156]. The amplification of neutrophil infiltration and NET formation in post-MI apoE^−/−^ mice significantly increased inflammatory and oxidative damage in the myocardial infarct zone [154]. Neutrophilia in hypokalaemic mice is also correlated with a significantly higher risk of ventricular tachycardia post-MI, indicating that the detrimental role of neutrophils is not only restricted to the initial inflammatory response, but also contributes to the electrophysiological complications of MI [157]. Thus, the above studies support the notion that excessive neutrophil responses are damaging during both the early ischaemic and reperfusion phases of a MI.

It is, however, important to note that despite the evidence implicating neutrophils as mediators of cardiac damage and dysfunction after MI, recent studies have reported that neutrophils can also exert protective functions within the healing myocardium. For example, a landmark study by Ma et al. identified N1 (pro-inflammatory) and N2 (anti-inflammatory) polarised neutrophil subsets in a mouse MI model [158]. The N1 neutrophil subset accounted for 80% of all leukocytes in the initial infarct area. However, the proportion of N2 neutrophils increased from 2% on day 1 to 18% on day 7 post-MI, [158] and was accompanied by increased gene expression of anti-inflammatory markers, such as IL-10 [158]. This study highlights that there is plasticity of neutrophil function and phenotype following a MI, shifting from an initial pro-inflammatory NI to an N2 anti-inflammatory phenotype when the healing process and scar formation begins. This finding also supports that manipulation of N1 versus N2 neutrophil phenotype at the optimal time is a potentially novel approach for attenuating LV remodelling and improved myocardial healing [128]. 

Other beneficial roles of neutrophils include their capacity to promote angiogenesis, which is necessary for successful myocardial revascularisation and the repair of tissue damage after a MI [159]. For example, the deposition of neutrophil-derived cathelicidin, an anti-microbial polypeptide with chemotactic activity, along the endothelium of injured arteries activates circulating endothelial precursor cells [160]. This suggests that neutrophils may contribute to beneficial endothelial repair in patients with CAD. Furthermore, studies in neutrophil-deficient mice have reported significantly reduced levels of tissue-reparative anti-inflammatory M2 macrophages in areas of cardiac damage post-MI [161,162]. Thus, blanket elimination of neutrophils may not necessarily constitute an effective strategy for MI treatment, as their protective anti-inflammatory functions and crosstalk with reparative endothelial progenitor cells and M2 macrophages would be lost. Instead, strategies that selectively target the tissue-damaging pro-inflammatory and oxidative actions of neutrophil-derived granular enzymes such as MPO will likely be a more appropriate and effective approach for reducing post-MI myocardial damage. 

### 5.2. Clinical Evidence Correlating MPO with Post-MI Complications

The following sections discuss the clinical and experimental evidence for neutrophil-derived MPO as a significant enzymatic source of tissue damaging oxidants capable of promoting post-MI cardiac damage and dysfunction and the longer-term onset of HF [163,164,165,166,167].

Plasma MPO levels peak rapidly after MI onset, supporting that the activation and degranulation of circulating neutrophils is an early event, implicating a role for MPO in post-MI cardiac complications [91]. Consistent with this, increased plasma MPO in MI patients undergoing primary PCI independently predicts increased in-hospital mortality and subsequent clinical complications [168], including ventricular arrhythmia, which is the leading acute cause of death post-MI. MI patients with arrhythmic events including ventricular arrhythmia, sudden cardiac death, or cardioverter-defibrillator implantation, have significantly higher circulating MPO levels than those without arrhythmic events [169]. Increased circulating MPO levels in MI patients at the time of admission are associated with increased in-hospital rates of arrhythmias, as well as re-infarction and death [170]. These studies provide evidence that MPO increases myocardial susceptibility to ventricular arrhythmias post-MI. 

Cardiogenic shock is another feared acute complication of MI. Clinical data links elevated circulating MPO levels with increased risk of death in patients that develop cardiogenic shock shortly after MI [171]. In a small study examining 38 consecutive patients being treated with primary PCI for STEMI complicated by cardiogenic shock, 20 patients died in the coronary care unit [171]. When compared to the patients that survived, those who died had significantly higher circulating MPO levels [171]. 

Recent studies have focused on the potential for MPO as a predictor of long-term survival and delayed adverse cardiac outcomes in MI survivors. Cohort studies have established that elevated circulating MPO levels independently predict increased mortality after reperfusion in MI survivors, with follow-up times ranging from 6 months to over 13 years [53,56,57,172,173]. Elevated MPO levels in the days following a MI can also be an independent predictor of future major adverse cardiovascular events (MACE), including stroke, MI, and HF leading to hospitalisation and cardiovascular death [62,173,174]. This trend remains even in patients undergoing an elective coronary angiography, with two studies demonstrating that plasma MPO levels > 322 pmol/L significantly increased the risk of future acute MACE [58,175]. Therefore, it is evident that an elevated plasma MPO levels is a potential predictor for adverse cardiovascular outcomes in MI survivors.

Increased circulating MPO levels are also associated with adverse cardiac remodelling and future HF in MI survivors, implicating a role for MPO in the long-term adverse effects of MI. A 2021 prospective cohort study of MI patients who underwent PCI found that those with above-median levels of MPO within intravascular thrombi had significantly increased adverse LV remodelling and reduced LVEF after one month [176]. 

Circulating MPO levels remain persistently elevated in MI patients that subsequently develop HF [62]. In cohort studies of patients with chronic HF, plasma MPO levels were significantly elevated compared to the control patients [177]. These findings indicate that although MPO is largely released in the acute inflammatory stages post-MI, persistent elevation of this enzyme is linked to the development of longer-term adverse clinical cardiovascular outcomes. These outcomes can appear months to years after the MI and manifest as progressively reduced cardiac function and survival [101]. 

Elevated MPO activity has been additionally linked to worse LV function in long-term stable cardiac patients. One study found that MPO plasma levels were significantly elevated in a cohort of patients with impaired LV function [178]. The discovery that the -463G/A promoter polymorphism in the MPO gene regulates transcription, with the G allele being linked to increased protein expression, led to the question of whether this polymorphism is predictive of the prognosis of cardiac patients [178,179]. However, no association between the MPO-463 promoter polymorphism and LV dysfunction was reported [178]. A subsequent study examining the clinical outcomes of MI patients with LV dysfunction, separated into cohorts depending on their -463G/A promoter polymorphism [179], found no relationship between the polymorphism and MPO plasma levels, but the GG genotype was associated with a significant decrease in survival over 1050 days [179]. This clinical evidence highlights that MPO activity within the myocardium can worsen LV dysfunction for MI survivors.

### 5.3. Preclinical Evidence for MPO as a Driver of Cardiac Damage and Complications Post-MI

Various pre-clinical studies in animal models of MI and in vitro cell culture studies have provided further support for the detrimental actions of MPO on the myocardium post-MI. There is good evidence for elevated MPO levels and the accumulation of MPO-oxidised lipids and proteins in the myocardium and infarct zone during the reperfusion phase post-MI [68,163,180]. For example, the accumulation of α-chloro fatty aldehydes, that are produced by MPO-derived HOCl has been reported in rat cardiac tissue post-MI [181]. Neutropenic rat hearts also have significantly decreased levels of α-chloro fatty aldehydes, highlighting the importance of neutrophil-derived MPO in the oxidative modification of lipids within the infarcted myocardium [181]. Myocardial levels of 3-chlorotyrosine, a selective protein biomarker for the production of MPO-derived HOCl, and MPO-oxidised myoglobin were also elevated in rat hearts within 24 h post-MI when compared to the control and sham groups [163]. This indicates that MPO mediates oxidative myocardial tissue damage in the acute stages post-MI that may contribute to the chronic cardiac dysfunction seen in the longer-term cohort studies. 

To date, most pre-clinical evidence has concluded that MPO contributes to adverse LV remodelling and dysfunction, but not to overall infarct size in experimental MI. This suggests that MPO-mediated oxidative damage primarily impairs cardiomyocyte function, resulting in future adverse cardiac outcomes, rather than mediating excessive cardiomyocyte death. Vasilyev et al. were the first to document this in a murine MI model of transient left anterior ascending (LAD) coronary artery ligation followed by reperfusion [182]. In that study, MPO-mediated oxidative tissue damage in the myocardium post-MI in wild-type mice coincided with the development of LV dysfunction. In comparison, while MPO gene-knockout mice had a similar infarct size as wild-type mice, they had significantly less LV remodelling and dysfunction 24 days post-MI [182]. 

Another key study that examined the effects of MPO during persistent myocardial ischaemia in response to permanent LAD coronary artery ligation reported similar findings with MPO gene-knockout mice displaying significantly improved LV remodelling and function 21 days post-MI compared to wild-type mice, despite similarities in infarct size [183]. In a more recent study, Mollenhauer et al. employed the transient LAD ligation I/R MI model to demonstrate that MPO gene-deficient mice had improved LV function compared to wild-type mice, and that this correlated with a significant reduction in ventricular fibrosis 7 days post-MI [169]. Notably, MPO gene-deficient mice showed a reduced slowdown and heterogeneity of electrical conduction in the peri-infarct zone relative to wild-type mice 21 days post-MI, which supports a role for MPO as a mediator of ventricular arrhythmias [169]. As before, there was no difference in infarct size between the two groups, providing further pre-clinical evidence that MPO-mediated oxidative myocardial tissue damage primarily impairs the functionality of the remaining viable cardiomyocytes.

In addition to MPO’s role as a mediator of LV electrical disturbances during MI, MPO also plays a role in promoting atrial fibrosis and fibrillation. In an angiotensin II-induced inflammatory mouse model of atrial fibrillation, MPO-deficient mice exhibited a signification reduction in atrial tissue levels of HOCl, active MMPs, and atrial fibrosis compared to wild-type mice [184]. Furthermore, upon cardiac electrophysical stimulation, the MPO-deficient mice exhibited reduced atrial fibrillation, which was reversed by chronic intravascular infusion of human neutrophil-derived MPO [184]. Atrial fibrillation patients also have increased circulating and atrial tissue levels of MPO and elevated atrial levels of 3-chlorotyrosine, which indicates MPO-mediated oxidative tissue damage [184]. Table 1 summarises recent studies investigating the role of MPO in post-MI or arrhythmia pre-clinical animal models. 

While the above animal studies provide important insights into the role of MPO in MI, the extent to which these findings translate to the human clinical context requires further investigation. This is because mouse neutrophils contain 20–30% of the MPO levels of human neutrophils [185] and experimental MI models are commonly performed in relatively young mice without established CAD or other cardiovascular risk factors (e.g., hypercholesterolemia, diabetes, hypertension), which is commonly the case with older human MI patients.

**Table 1:** Impact of MPO interventions in pre-clinical models of MI or arrhythmia.

Pre-Clinical Model of MI or Arrhythmia	MPO Intervention	Impact of Intervention on Study Outcome	Reference
Permanent LAD ligation in rats	Pre-treatment with anti-neutrophil antibody	Diminished α-chloro fatty acid aldehydes and neutrophil infiltration levels in the infarcted myocardium	Thukkani, A.K., Martinson, B.D., Albert, C.J., Vogler, G.A. & Ford, D.A., 2005 [181]
Temporary (ischemia-reperfusion model) LAD ligation in rats	MPO knockout genotype	Reduced LV dilation and improved LV function, although no difference in infarct size	Vasilyev et al., 2005 [182]
Permanent LAD ligation in mice	MPO knockout genotype	Decreased leukocyte infiltration, reduced LV dilation and preserved LV function	Askari et al., 2003 [183]
Both permanent and temporary (ischemia-reperfusion model) LAD ligation in mice	MPO knockout genotype	Decreased vulnerability for ventricular tachycardia and decreased heterogeneity of electrical conduction in the peri-infarct zone	Mollenhauer et al., 2017 [169]
Permanent LAD ligation in mice	Treatment with MPO inhibitor PF-1355	Improved ejection fraction, decreased end diastolic volume and LV mass	Ali et al., 2016 [186]
Temporary (ischemia-reperfusion model) LAD ligation in mice	Treatment with MPO inhibitor AZM198	Improved cardiac function and reduced structural remodelling	Guthoff et al., 2022 [187]
Right atrial electrophysical stimulation in mice	MPO knockout genotype	Protection from atrial fibrillation	Rudolph et al., 2010 [184]

### 5.4. Proposed Mechanisms by Which MPO Mediates Cardiac Damage and Complications Post-MI

**Figure 3:**
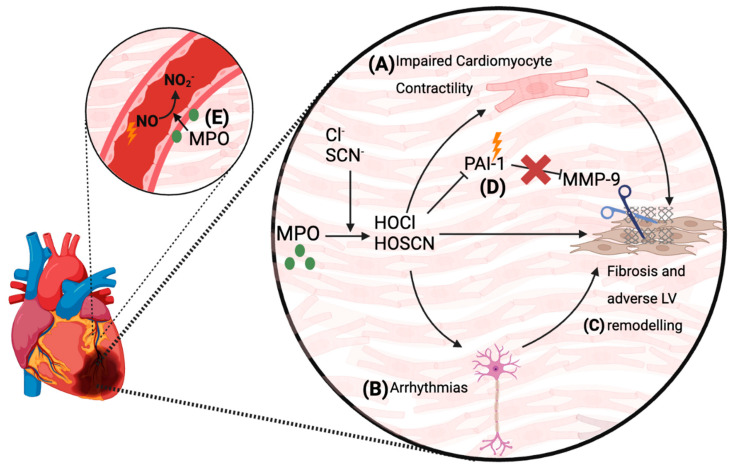
**Pathophysiological actions of MPO in the myocardium and the endothelium post-MI.** Both clinical and experimental data have documented the increased levels of MPO and its oxidants within the myocardium post-MI. Several mechanisms for how MPO adversely impacts LV remodelling and function have been proposed. (**A**) MPO-mediated oxidation of myofilament proteins in cardiomyocytes can impair their contractility [188]. (**B**) MPO-derived oxidants can impair the myocardial electrical conducting system, and can cause arrhythmias [169]. (**C**) Impaired cardiomyocyte contractility and electrical conduction pathways, as well as the direct pro-inflammatory effects of MPO-derived oxidants, can lead to long-term LV fibrosis and adverse remodelling, with a chronic decline in cardiac function years after the MI [169,182]. (**D**) Plasminogen activator inhibitor-1 (PAI-1) is inhibited by MPO-mediated oxidation. This facilitates MMP activation leading to adverse LV remodelling. (**E**) Accumulation of MPO in the endothelium of infarct-related coronary arteries restricts myocardial perfusion post-MI by consuming NO and promoting endothelial dysfunction [48].

The current understanding of the molecular mechanisms by which MPO-mediated oxidation leads to cardiomyocyte dysfunction and the subsequent adverse cardiac outcomes post-MI is incompletely understood. However, in vivo and in vitro works in mouse MI models and cultured cardiomyocytes have provided some key insights. Askari and colleagues have reported that plasminogen activator inhibitor-1 (PAI-1) is an in vivo target of MPO-derived oxidants in the infarcted heart, resulting in PAI-1 inactivation, activation of MMPs, and impaired LV function [183] (Figure 3). In vitro studies indicate that exposing cardiomyocytes to MPO-derived oxidants impairs their contractile function by oxidizing myofilament proteins and reducing the maximal calcium-dependent force [188], which may contribute to MPO-dependent LV dysfunction post-MI (Figure 3). Furthermore, when cardiomyocytes were exposed to the MPO-derived oxidants HOCl and HOSCN in vitro, there was a significant dose-dependent loss of mitochondrial inner trans-membrane potential, which also has a detrimental effect on cardiomyocyte function [189]. These findings provide some molecular insights into how MPO promotes LV dysfunction and future heart failure, although more detailed mechanistic work is warranted in this area [176]. 

A further post-MI complication is coronary artery and microvascular endothelial dysfunction resulting in impaired myocardial perfusion that is exacerbated by inflammation [190,191]. These complications are accompanied by the accumulation of MPO into infarct-related coronary arteries [70], which has the ability to catalytically consume NO and impair endothelial function [75]. For example, plasma from re-vascularised MI patients with increased levels of MPO showed enhanced consumption of NO in the presence of the MPO co-substate H_2_O_2_ [48]. Moreover, in patients with symptomatic CAD, the extent of increase in plasma MPO levels correlated with the degree of microvascular endothelial dysfunction [48]. Thus, the accumulation of MPO into the coronary vasculature and resultant impairment of NO bioactivity and endothelial function can conceivably contribute to the compromised coronary circulation function and poor myocardial perfusion in MI survivors (Figure 3) [48,62,70,101]. 

## 6. MPO as a Therapeutic Target for the Treatment of Cardiac Dysfunction Post-MI

The overall clinical, experimental animal, and in vitro evidence pointing to the detrimental vascular and cardiac effects of MPO identify this enzyme as a promising therapeutic target with the potential to improve the clinical outcomes of MI survivors. In the last two decades, there has been considerable global interest in the discovery and development of pharmacological therapeutics that selectively and potently target MPO for treating a variety of inflammatory disorders. Importantly, while MPO contributes to the innate immune defence against infection, MPO-deficient humans and mice are resistant to life-threatening opportunistic infectious agents, with the exception of specific fungal pathogens [192]. These findings support that the pharmacological inhibition of MPO is therapeutically and clinically viable without increasing the risk of infection, as has been reported for broad spectrum anti-inflammatory agents in at-risk CAD patients in the recent CANTOS and COLCOT randomised clinical trials [193,194]. 

Two principal pharmacological approaches exist to inhibit MPO’s catalytic activity: (1) irreversible enzyme inactivation by permanent covalent bonding of pharmacological agents to the haem active site and (2) reversible inhibition with competitive substrates that bind temporarily to the active site and transiently suppress activity [195]. 

Irreversible MPO inhibition has received considerable recent attention as a potent and clinically viable option and has been used in several recent clinical trials. In particular, 2-thioxanthines are a topical class of small-molecule irreversible inhibitors currently being investigated in pre-clinical and clinical studies [41]. Mechanistically, 2-thioxanthines are initially rapidly oxidised by MPO compound I into a free radical, which in turn irreversibly binds to MPO compound II, thereby converting the enzyme into an inactive form (Figure 1E,F) [41]. Thiouracil derivatives are another class of irreversible inhibitor compounds with a similar mechanism of action to the 2-thioxanthines [41]. 

To date, two different 2-thioxanthine inhibitors have been tested in phase 1 clinical trials for the treatment of neuro-inflammatory diseases, including multiple systems atrophy (AZD3421) and multiple sclerosis (AZD5904) [41]. Preliminary results show that irreversible MPO inhibition is safe in humans and reduces inflammatory activity [196]. AZD5904 was also studied in phase 1 clinical trials for the treatment of chronic obstructive pulmonary disease (COPD) [41]. However, none of the compounds have been developed beyond phase II clinical trials as their pharmacological properties were not superior to existing treatment options for these conditions [196]. 

In the context of cardiovascular disease, irreversible pharmacological MPO inhibitors have been tested pre-clinically and more recently progressed to clinical trials in heart failure patients. In pre-clinical studies, irreversible MPO inhibitors have been used in several animal models of cardiovascular disease including MI. For example, irreversible MPO inhibition with PF-06282999 did not significantly affect lesion area in atherosclerosis-prone LDL receptor gene-knockout mice fed a high fat Western diet for 14 weeks. However, it did reduce necrotic core size in advanced aortic root atherosclerotic lesions [197], suggesting that MPO inhibition may promote lesion stability. Importantly, in that study the MPO inhibitor and high fat diet were given concurrently and hence the treatment was present throughout the initiation and progression phases of disease. As CAD patients present clinically with established atherosclerosis, it is important for further studies to examine the impact of MPO inhibitor drugs on the ongoing development and stability of already established atherosclerotic lesions. The tandem stenosis model of atherosclerotic plaque instability in apoE^−/−^ mice has also shown that treatment with the 2-thioxanthine AZM198 effectively inhibited MPO activity in pre-existing unstable atherosclerotic lesions and increased fibrous cap thickness without affecting the levels of lesion monocytes or circulating leukocytes and lipids [90,198]. AZM198 therapy also reduced endothelial dysfunction in the femoral cuff and tandem stenosis murine models of vascular disease [199]. The benefits of MPO inhibition may also extend beyond the cardiovascular system. MPO inhibition with AZM198 in obese mice that were fed a high fat diet for 16 weeks with the last 4 weeks accompanied by angiotensin-II infusion to induce hypertension had significantly reduced visceral adipose tissue inflammation and non-alcoholic steatohepatitis [200]. However, cardiac hypertrophy, poor cardiac contractility and hepatic fibrosis were not ameliorated by AZM198 treatment in these obese, hypertensive mice [200]. 

Two preclinical trials of irreversible MPO inhibition for the treatment of MI have been conducted. In one study, Ali et al. showed that treatment with PF-1355, an irreversible orally available MPO inhibitor, significantly decreased the number of inflammatory cells present within the myocardium and attenuated LV dilation 7 days post-MI. It also improved LVEF and decreased LV end-diastolic volume after 21 days [186]. MPO inhibition did not, however, decrease infarct size at two days post-MI. Importantly, as this MI model used a permanent surgical ligation of the LAD coronary artery, the ligated artery was not reperfused. As MPO is prominent during reperfusion of the infarcted myocardium, and as most MI patients undergo reperfusion therapy by PCI, further pre-clinical trials studying the use of MPO inhibitors in models of reperfusion are required to provide additional insights into the therapeutic potential of MPO inhibition in MI. To date, only one pre-clinical study has addressed this. Guthoff et al. treated mice with 21 days of the 2-thioxanthine AZM198 post-MI and reperfusion of the myocardium after 40 min of LAD coronary artery ligation [187]. Trichome staining of murine hearts 21 days after the ischaemia-reperfusion injury showed significantly less LV fibrosis in AZM198-treated mice compared to the control mice [187]. Pressure-volume loop analyses also revealed significantly improved LV ejection fraction and cardiac output in the AZM198-treated mice [187].

Despite these promising preclinical results, it is important to note that several barriers exist that prevent translation of the preclinical findings into clinical practice. As noted above, the experimental animals are young, bred in a tightly controlled sterile environment, and do not have any cardiovascular comorbidities prior to surgical induction of MI. In humans, MI is preceded by decades of cholesterol deposition within the arterial intima leading to atherosclerosis, plaque rupture, and eventual thrombotic occlusion of coronary artery blood flow [6].

In a cohort study of 69,571 men and 38,930 women undergoing PCI for MI, 95.5% of patients had at least one conventional cardiovascular risk factor for CAD, while approximately half of the patients had at least three risk factors [201]. The conventional cardiovascular risk factors included cigarette smoking, hypertension, dyslipidaemia, diabetes, and obesity. These comorbidities lead to the adverse lipid profiling and exert other effects that may potentiate MI and influence myocardial healing [128]. These effects are not observed when a MI is induced by surgical ligation of a coronary artery in animal models. Future studies on the role of MPO post-MI should ideally be conducted in animals with one or more of these cardiovascular risk factors that predispose to CAD [186]. It is important to note that in order to better capture the true clinical picture of humans presenting with MI, the effects of ageing in animal models should also be studied, as survival rates significantly decrease with age after surgical MI induction [202,203]. Moreover, the efficacy of cardioprotective treatments may also be altered in aged mice [204]. 

To date, there are no clinical trials examining the effects of MPO inhibition in patients with unstable CAD or recent MI. However, the 2-thioxanthine AZD4831 has recently progressed to clinical trials for the treatment of heart failure patients with a preserved ejection fraction (HFpEF) [205,206,207]. Many of the mechanisms underlying the pathogenesis of HFpEF remain elusive and, with limited treatment options, treatment of the condition is a significant unmet need in cardiovascular medicine [208]. While prior studies have established that circulating MPO levels are enhanced in patients that have chronic heart failure with a reduced ejection fraction (HFrEF), which predicts increased disease severity and risk of adverse clinical outcomes [177,209], a potential role for MPO in promoting the microvascular inflammation and dysfunction during HFpEF has only been recognised recently. Thus, Hage et al. indicated that MPO levels are elevated in patients with HFpEF, and that they are correlated with biomarkers of neutrophil activation, inflammation, and endothelial dysfunction [208]. Based on pre-clinical and clinical data supporting MPO’s role as a key mediator of cardiovascular oxidative stress that elicits microvascular and myocardial dysfunction [74,169,182,184], clinical studies have explored the utility of AZD4831 as a novel treatment of HFpEF. Initial pharmacokinetic analysis of AZD4831 showed that it is well-tolerated, rapidly absorbed, and has a long plasma half-life in healthy men, making it suitable for once-daily oral administration [210,211,212]. AZD4831 also has strong selectivity for MPO inhibition over thyroid peroxidase, as well as limited penetration of the blood–brain barrier [213]. These factors identified AZD4831 as a suitable candidate for further clinical trials. In 2023, a double-blinded phase 2a study (Safety and Tolerability Study of AZD4831 in Patients With Heart Failure [SATELLITE]) in 41 patients with symptomatic heart failure with a LVEF of ≥40% were subjected to either a once-daily 5 mg oral dose of AZD4831 or a placebo [205]. Due to the COVID-19 pandemic, the study findings ended up only being exploratory, but AZD4831 was found to inhibit MPO and was well tolerated in this patient cohort. There was also a trend towards improvement in scores of the Kansas City Cardiomyopathy Questionnaire in the patients treated with AZD4831 [205]. Following on from this, in a study in which numerous plasma biomarkers and clinical outcomes were determined in three independent HFpEF cohorts (n = 86, n = 216, n = 242), they were compared to the profiles of the patients in the SATELLITE study [207]. Pathophysiological pathways that were inferred from the biomarker profiles and found to be implicated in heart failure hospitalisation or death were associated with tumour microenvironments, wound healing signalling, and cardiac hypertrophy signalling [207]. Importantly, these pathways were also down-regulated in patients treated with AZD4831 [207]. Currently, AZD4831 is being studied in a sequential phase 2b-3 randomised, double-blind, parallel-group, placebo-controlled clinical trial (ENDEAVOR) to evaluate its effect on symptoms and exercise tolerance on patients with HFpEF (EF > 40%) over 48 weeks [206]. 

Finally, hypertrophic cardiomyopathy (HCM) is a common cause of HFpEF [214]. Human cardiomyocytes derived from control-induced pluripotent stem cells (iPSC-CMs) express MPO, which was significantly increased in two HCM patients [214]. Furthermore, treatment with an irreversible MPO inhibitor alleviated cardiomyocyte dysfunction in hypertrophic iPSC-CMs [214]. These results support the further investigation of pharmacological MPO inhibition as a treatment option for HFpEF. 

## 7. Peroxidasin and MI

MPO is a member of a broader family of mammalian haem peroxidase enzymes. While most of the focus on haem peroxidase enzymes in cardiovascular disease has been on MPO, mounting evidence indicates that another family member, peroxidasin, can also impact disease progression and severity. Peroxidasin is highly expressed in endothelial cells, smooth muscle cells and cardiomyocytes [215]. Due to its high expression in the vasculature peroxidasin has also been called vascular peroxidase 1 (VPO1), which, like MPO, produces HOBr and HOCl [215,216,217]. Under physiological conditions, peroxidasin generates these hypohalous acids to facilitate the generation of sulphilimine bonds between adjacent collagen IV protomers, thereby contributing to the formation of cross-linked collagen networks that are required to establish stable basement membrane structures [218,219]. However, peroxidasin/VPO1 is upregulated in vivo in inflamed or diseased cardiovascular tissue including human and mouse abdominal aortic aneurysms [220], atherosclerotic lesions in apoE^−/−^ mice [221] and in response to pathogenic stimuli, including angiotensin II [222,223]. Notably, peroxidasin/VPO1 has a pathogenic role in a mouse model of MI through elevating cardiac fibrosis [224]. It also promotes pathogenic events that are important in inflammatory artery disease, including promoting vascular oxidative stress, endothelial dysfunction [225,226], activation of MMPs, and the induction of phenotypic switch of SMC from a contractile to a synthetic phenotype [220]. Therefore, it is plausible that in addition to MPO, the upregulation of peroxidasin/VPO1 may also contribute to the pathogenesis of CVD and post-MI cardiac inflammation and dysfunction. Interestingly, 2-thioxanthines, as a class of clinically viable MPO inhibitors, can inhibit peroxidasin activity, albeit at 20-fold reduced efficacy compared to MPO [227]. Therefore, the development of inhibitors capable of targeting both MPO and peroxidasin represents a potentially promising avenue of investigation with respect to effectively targeting peroxidase-mediated pathology in atherosclerosis and MI. However, more pre-clinical and clinical studies are required before the respective contributions of MPO and peroxidasin can be gauged.

## 8. Conclusions and Future Directions

This review identifies MPO as a critical mediator of inflammatory and oxidative processes in CAD and MI. Consequently, there is growing interest in MPO as a therapeutic target for treating patients with CAD or MI. Accordingly, MPO gene-deficiency and inhibition has been reported to protect against atherosclerotic plaque instability and MI in pre-clinical models of cardiovascular disease [186,187,197,198,199]. However, while MPO gene deficiency protects against LV dysfunction post-MI [182,183], only one pre-clinical study by Guthoff and colleagues has examined the utility of MPO inhibition in a MI model with reperfusion [187]. Further pre-clinical work that utilises the myocardial ischaemia-reperfusion model and includes relevant comorbidities and aged mice would further our understanding of the applicability of MPO inhibition to the acute clinical treatment of post-MI cardiac dysfunction.

In the clinical setting, MPO inhibition with the 2-thioxanthine AZD4831 is being evaluated for the treatment of HFpEF in a phase III clinical trial [205,206,207]. To date, AZD4831 exhibits a favourable pharmacokinetic profile [210,211,212] and downregulates pathophysiological pathways that are closely associated with HF hospitalisation or death [207]. Therefore, MPO inhibition holds promise as a viable clinical treatment of a sub-set of HF patients. Considering the data pointing to a pathological role of MPO in promoting plaque instability and post-MI cardiac complications, studies investigating whether pharmacological MPO inhibition can also attenuate the risk of CAD and ACS patients or improve cardiovascular remodelling and dysfunction in MI survivors are warranted.

As MPO accumulates into the coronary endothelium in CAD patients and MI survivors [96], a further therapeutic option is the removal of endothelial-sequestered MPO from the coronary endothelium back into the circulation with heparins [228]. The use of heparins to remove MPO from diseased cardiovascular tissue may also protect against MPO’s non-catalytic pro-inflammatory actions that may conceivably contribute to pathogenic actions [40]. Although the role of MPO’s non-catalytic inflammatory activities in atherosclerotic cardiovascular disease and MI are unknown, if important, these non-enzymatic actions will be refractory to MPO inhibitors such as 2-thioxanthines, which may make alternative treatment with heparins necessary to completely neutralise MPO’s pathogenic actions [40].

A further area of interest is the potential role of peroxidasin in post-MI complications. As MPO and peroxidasin are both implicated as causing cardiac dysfunction post-MI in pre-clinical models, future work should consider the relative contributions of both enzymes in MI. This may inform the development of potent MPO and peroxidasin dual inhibitors as novel therapeutics for treating MI patients. Similarly, inhibition of other sources of oxidative stress that drive post-MI complications in conjunction with peroxidase inhibition may also prove beneficial. For example, mitochondrial ROS play a role in cardiomyocyte dysfunction and death post-MI, and mitochondrial-targeted antioxidants have been shown to protect against this in relevant pre-clinical models [229,230,231,232]. Thus, mitochondrial-targeted therapeutics also hold promise in the clinical treatment of cardiovascular disorders including heart failure [232,233].

The translation of MPO-targeted therapeutics for the treatment of MI survivors also requires an understanding of the optimal timeframe of treatment. This is because there is an increased awareness of the complex temporal dynamics of the inflammatory responses that occur post-MI, including dynamic changes in inflammatory and immune cell numbers and phenotypes in the myocardium over the days and weeks following a MI [128]. Accordingly, a better understanding of the timeframe during which neutrophils and MPO exert their beneficial and detrimental actions post-MI will aid the development of effective therapeutic regimens. Currently, neutrophils and MPO are implicated as playing a role during the acute inflammatory phase of healing (hours to days) post-MI [128,141]. Therefore, MPO inhibitor treatment commenced immediately post-MI may result in better cardiac outcomes for MI patients. As indicated earlier, however, elucidating how reperfusion also impacts on the efficacy and timing of MPO-targeted therapies requires further understanding. Therefore, while targeting MPO represents a promising option for treating the initial myocardial complications in MI survivors, further pre-clinical and clinical studies are needed before MPO inhibitors can be introduced safely into clinical settings.

## Data Availability

Not applicable.
